# Molecular Characterization of Subtype H11N9 Avian Influenza Virus Isolated from Shorebirds in Brazil

**DOI:** 10.1371/journal.pone.0145627

**Published:** 2015-12-21

**Authors:** Renata Hurtado, Thomas Fabrizio, Ralph Eric Thijl Vanstreels, Scott Krauss, Richard J. Webby, Robert G. Webster, Edison Luiz Durigon

**Affiliations:** 1 Department of Preventive Veterinary Medicine and Animal Health, School of Veterinary Medicine, University of São Paulo, São Paulo, Brazil; 2 Division of Virology, Department of Infectious Diseases, St. Jude Children’s Research Hospital, Memphis, Tennessee, United States of America; 3 Laboratory of Wildlife Comparative Pathology, Department of Pathology, School of Veterinary Medicine, University of São Paulo, São Paulo, Brazil; 4 Laboratory Biosafety Level 3+, Department of Microbiology, Institute of Biomedical Sciences, University of São Paulo, São Paulo, Brazil; Linneaus University, SWEDEN

## Abstract

Migratory aquatic birds play an important role in the maintenance and spread of avian influenza viruses (AIV). Many species of aquatic migratory birds tend to use similar migration routes, also known as flyways, which serve as important circuits for the dissemination of AIV. In recent years there has been extensive surveillance of the virus in aquatic birds in the Northern Hemisphere; however in contrast only a few studies have been attempted to detect AIV in wild birds in South America. There are major flyways connecting South America to Central and North America, whereas avian migration routes between South America and the remaining continents are uncommon. As a result, it has been hypothesized that South American AIV strains would be most closely related to the strains from North America than to those from other regions in the world. We characterized the full genome of three AIV subtype H11N9 isolates obtained from ruddy turnstones (*Arenaria interpres*) on the Amazon coast of Brazil. For all gene segments, all three strains consistently clustered together within evolutionary lineages of AIV that had been previously described from aquatic birds in North America. In particular, the H11N9 isolates were remarkably closely related to AIV strains from shorebirds sampled at the Delaware Bay region, on the Northeastern coast of the USA, more than 5000 km away from where the isolates were retrieved. Additionally, there was also evidence of genetic similarity to AIV strains from ducks and teals from interior USA and Canada. These findings corroborate that migratory flyways of aquatic birds play an important role in determining the genetic structure of AIV in the Western hemisphere, with a strong epidemiological connectivity between North and South America.

## Introduction

Influenza A viruses are members of the family Orthomyxoviridae and contain 8 negative-sense RNA segments: polymerase basic protein 2 (PB2), polymerase basic protein 1 (PB1), polymerase acidic protein (PA), hemagglutinin (HA), nucleocapsid protein (NP), neuraminidase (NA), matrix protein (M), and nonstructural protein (NS) [1]. They are classified on the antigenic properties of HA and NA, two glycoproteins expressed on the virus surface. To date 18 HA and 11 NA subtypes have been identified, and these can be combined into a multitude of viral subtypes [2, 3]. In particular, H5 and H7 strains are considered the most relevant for the poultry industry due to their potential for high pathogenicity to chickens, whereas H1, H3, H5 and H7 strains have been documented to cause disease in humans, and recent H5N1 and H7N9 epidemics have resulted in significant human morbidity and mortality [4]. On the other hand, even though H11 strains have been shown to infect humans who have frequent contact with infected birds [5, 6], this subtype seems primarily associated with birds and has not been associated with signs of illness or mortality in any hosts [7–10].

Although occasionally leading to the death of wild birds [11–13], most infections by avian influenza viruses (AIV) in wild birds are subclinical or accompanied by mild clinical signs. Waterfowl and shorebirds are considered the major natural reservoirs of AIV, asymptomatically carrying a vast diversity of low pathogenicity avian influenza strains [13–15]. Although most of these strains do not have direct impacts for avian or human health, they serve as a greater gene pool from which highly pathogenic strains can emerge due to genetic re-assortments [16–18].

The ecology and genetic diversity of AIV has been widely studied in the Northern Hemisphere, with numerous studies demonstrating the patterns of gene flow among birds in different countries [13, 19, 20]. Migratory aquatic birds have been shown to play a key role in the international spread of AIV, and as a result the genetic diversity of AIV is reflective of the migratory routes of these birds [19, 20]. In particular, because many species of aquatic migratory birds tend to use similar migration routes, a number of “flyways” have been identified as major circuits for the spread of AIV [19, 21]. Most of these flyways have a latitudinal orientation and, as a result, the genome of AIV is generally distributed in two broad phylogenetic lineages, Eurasian and American, with limited gene exchange between them [20, 22]. Gene flow and persistence within the American lineage has been studied in depth, revealing that Anseriformes (ducks and teals) are determinant to the long-term persistence of AIV whereas migratory Charadriiformes (shorebirds) play a role in the short-term dissemination of the virus over long distances [23].

Even though the surveillance of aquatic birds for avian influenza virus has increased substantially worldwide in recent years, few studies have been conducted in South America [24, 25]. Considering there are major flyways connecting South America to Central and North Americas whereas avian migration routes between South America and the remaining continents are uncommon [19, 21, 26], it is fair to expect that AIV strains in this continent are most related to those from North and Central America. Only a handful of studies have studied the genome of AIV in wild birds in South America, with some strains being found to genetically resemble those from North America [17, 24, 27, 28] while others belong to distinct genetic clusters [29, 30].

In this study, we analyze the full genome of three H11N9 strains isolated from shorebirds on the Brazilian Amazon coast in a previous study [31]. We demonstrate that these viruses are related to North American strains obtained along the Atlantic, Mississippi and Pacific flyways, corroborating that migratory aquatic birds play a key role in the spread and gene flow of AIV within the Americas.

## Materials and Methods

The study was approved by the Ethics Committee on Animal Use of the School of Veterinary Medicine and Animal Science of the University of São Paulo (CEUA-USP 1752/2009), by the Brazilian Society of Laboratory Animal Science (Permit Number: 105, page 74, book 2) and by the Sistema de Autorização e Informação em Biodiversidade of the Instituto Chico Mendes de Conservação da Biodiversidade, Ministério do Meio Ambiente (SISBIO 21211–1, 25895–1).

Three AIV isolates from shorebirds in Northern Brazil were studied: A/ruddy turnstone/Ilha de Canelas/A008/2008(H11N9), A/ruddy turnstone/Ilha de Canelas/A017/2008(H11N9), and A/ruddy turnstone/Ilha de Canelas/A051/2008(H11N9). These isolates had been obtained from orotracheal and cloacal swabs of ruddy turnstones (*Arenaria interpres*) captured at Canelas Island (Ilha de Canelas; 0°47’7”S 46°43’23”W), within a region known as “Reentrâncias Paraenses”. These were the only AIV isolates obtained in a previous study [31] in which orotracheal and cloacal swabs from 556 aquatic birds captured at coastal areas of the Brazilian Amazon were tested for AIV by real-time RT-PCR, and virus isolation in embryonated eggs was attempted for PCR-positive samples.

Viral RNA was extracted from allantoic fluid with the MagMAX AI/NDV RNA extraction kit. All 8 RNA gene segments were reverse transcribed using SuperScript®III (Invitrogen, Life Technologies) following the recommended protocol. Following the reverse transcription all 8 gene segments were amplified using universal primers (Table 1) and Phusion polymerase (Thermo Fisher Scientific Products). The samples were then prepared for sequencing using the Nextera preparation kit (Illumina, Nextera XT DNA Library Preparation Guide, document # 15031942 Rev. E). Briefly, the amplified influenza gene segments were tagmented into about 300–500 base pair fragments which ligates the required Illumina adaptors. The tagmented fragments are then barcoded and purified using AMPure magnetic beads (Beckman Coulter). The enriched samples were then pooled and installed on the MiSeq instrument for cluster generation and sequencing. The resultant sequences were analyzed using CLC Genomics Workbench (Qiagen). The sequences of the segments were deposited on GenBank (accession numbers: KF824501-KF824506, KT932362-KT932379).

**Table 1:** Primer sequences.

Primer	Sequence (5’-3’)
Uni-12	AGCAAAAGCAGG
Uni-13	AGTAGAAACAAGG
PB2-1	AGCRAAAGCAGGTCAATTATATTCA
PB2-2341R	AGTAGAAACAAGGTCGTTTTTAAACTA
PB1-1	AGCRAAAGCAGGCAAACCATTTGAATG
PB1-2341R	AGTAGAAACAAGGCATTTTTTCATGAA
PA-1	AGCRAAAGCAGGTACTGATYCGAAATG
PA-2233R	AGTAGAAACAAGGTACTTTTTTGGACA

Influenza gene sequences were obtained from the Influenza Research Database website (IRD—http://www.fludb.org/) as available in 23 Sep 2015. All complete sequences of H11 and N9 were obtained; for the remaining segments, search criteria were selected to obtain complete segment sequences for the following groups: (1) avian strains worldwide, (2) H11N9 strains worldwide, (3) all strains from South America, (4) all strains from Antarctica, (5) avian strains from South America. Lineages that were incomplete or contained ambiguous nucleotide codes were excluded from the analysis; lineages retrieved from bats were also excluded. Sequences were aligned using the MUSCLE algorithm [32], and trimmed to the coding DNA sequences (CDS) using MEGA 6.06c [33]. Representative centroids were obtained using USEARCH 8.1.1756 [34], with varying levels of sequence identity (92–99%) being used to select approximately 350, 100 and 50 most representative centroids for sequence groups 1, 2 and 3, respectively. These representative centroids, along with the sequences from groups 4 and 5 and those obtained in this study, were used to construct maximum-likelihood trees for each of the viral genes using the GTR+G+I model (selected using jModelTest 2 [35]) as implemented in MEGA 6.06c, with 500 bootstrap replications.

MegaBLAST [36] was used to select the 50 GenBank sequences with highest identity to the CDS-trimmed sequences obtained in this study; this was done for each gene segment, resulting in a list of 400 high-identity sequences. This list was then used to determine high-identity sequence density for each state or province, i.e. the percentage of these 400 high-identity sequences that was recorded at that state or province. The migratory flyways [21, 37] and natural distribution of ruddy turnstones [38] were also represented.

## Results

**Fig 1:**
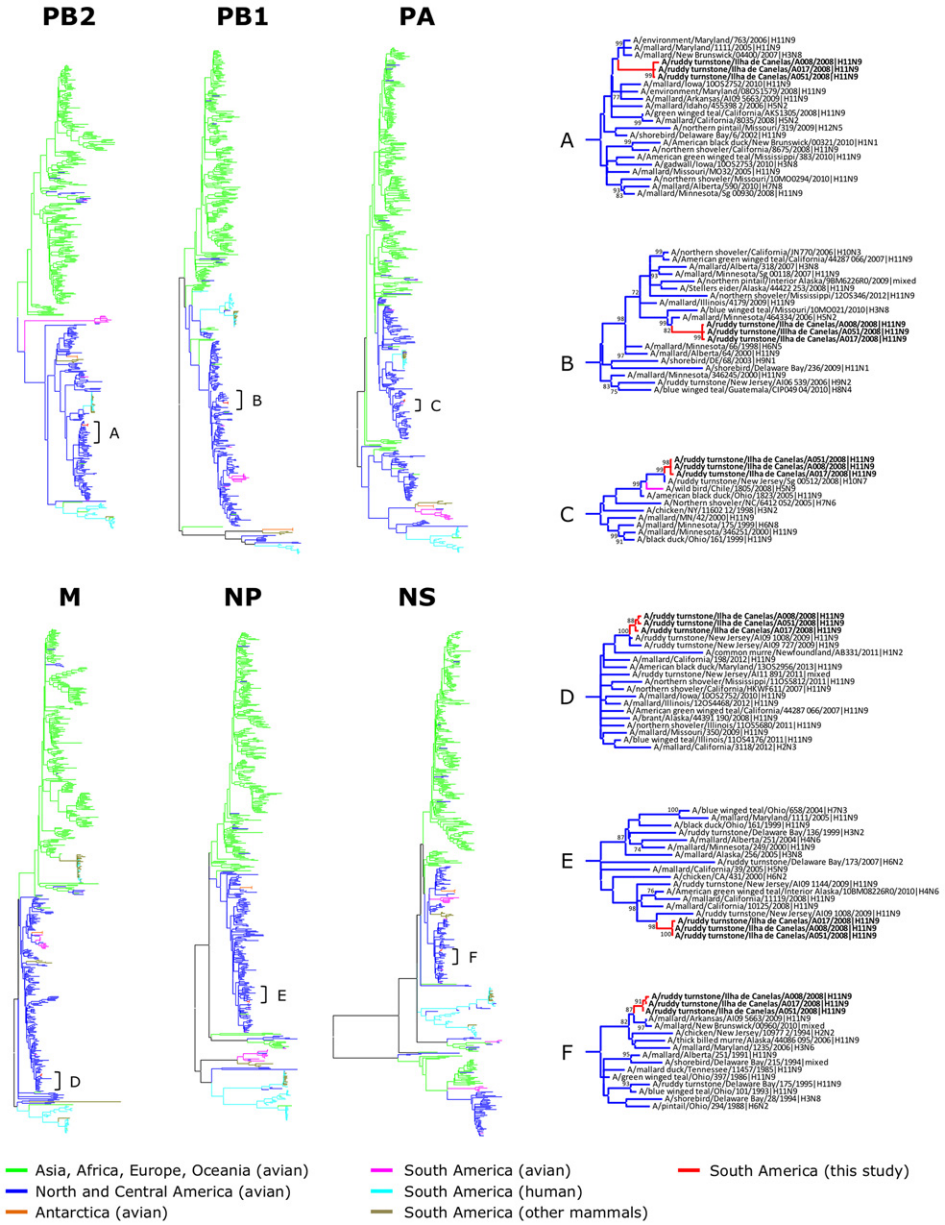
Phylogenetic tree of PB2, PB1, PA, M, NP and NS sequences. Trees are drawn to the similar scales, with branch lengths proportional to evolutionary distance. Bootstrap values lower than 30 are omitted.

**Fig 2:**
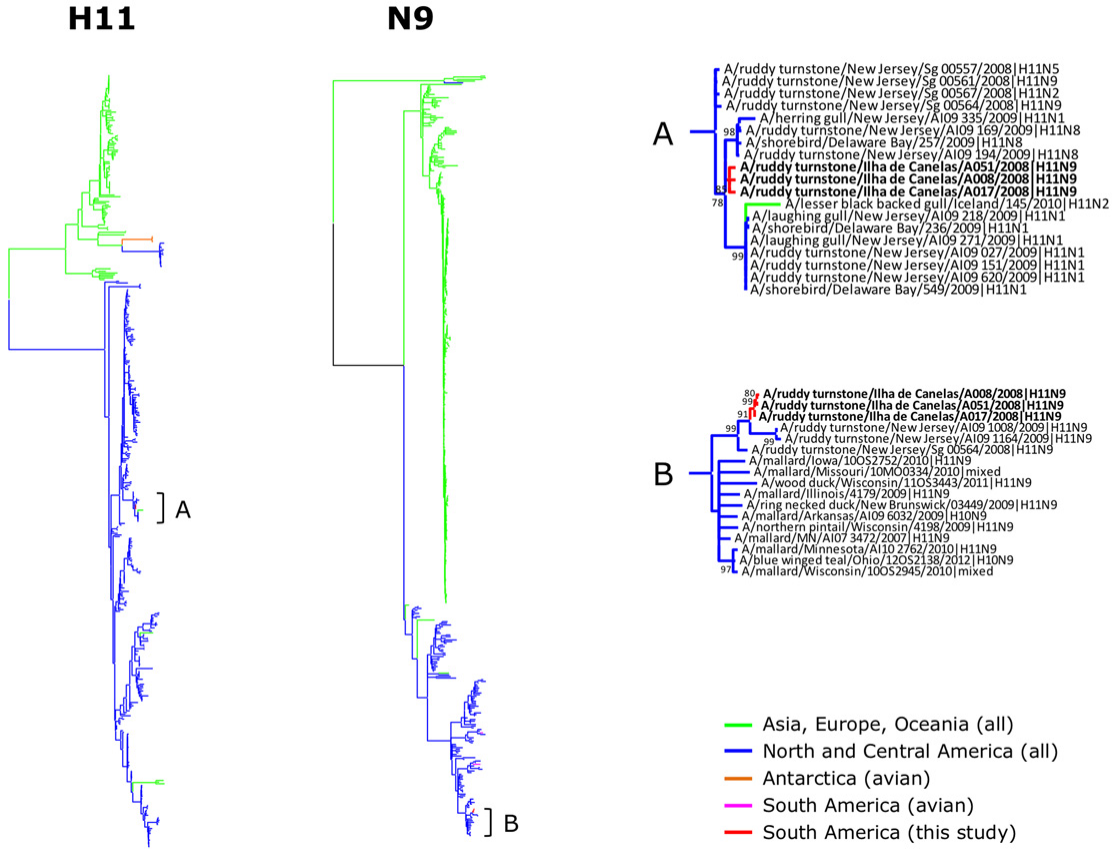
Phylogenetic tree of H11 and N9 sequences. Trees are drawn to the similar scales, with branch lengths proportional to evolutionary distance. Bootstrap values lower than 30 are omitted.

**Fig 3:**
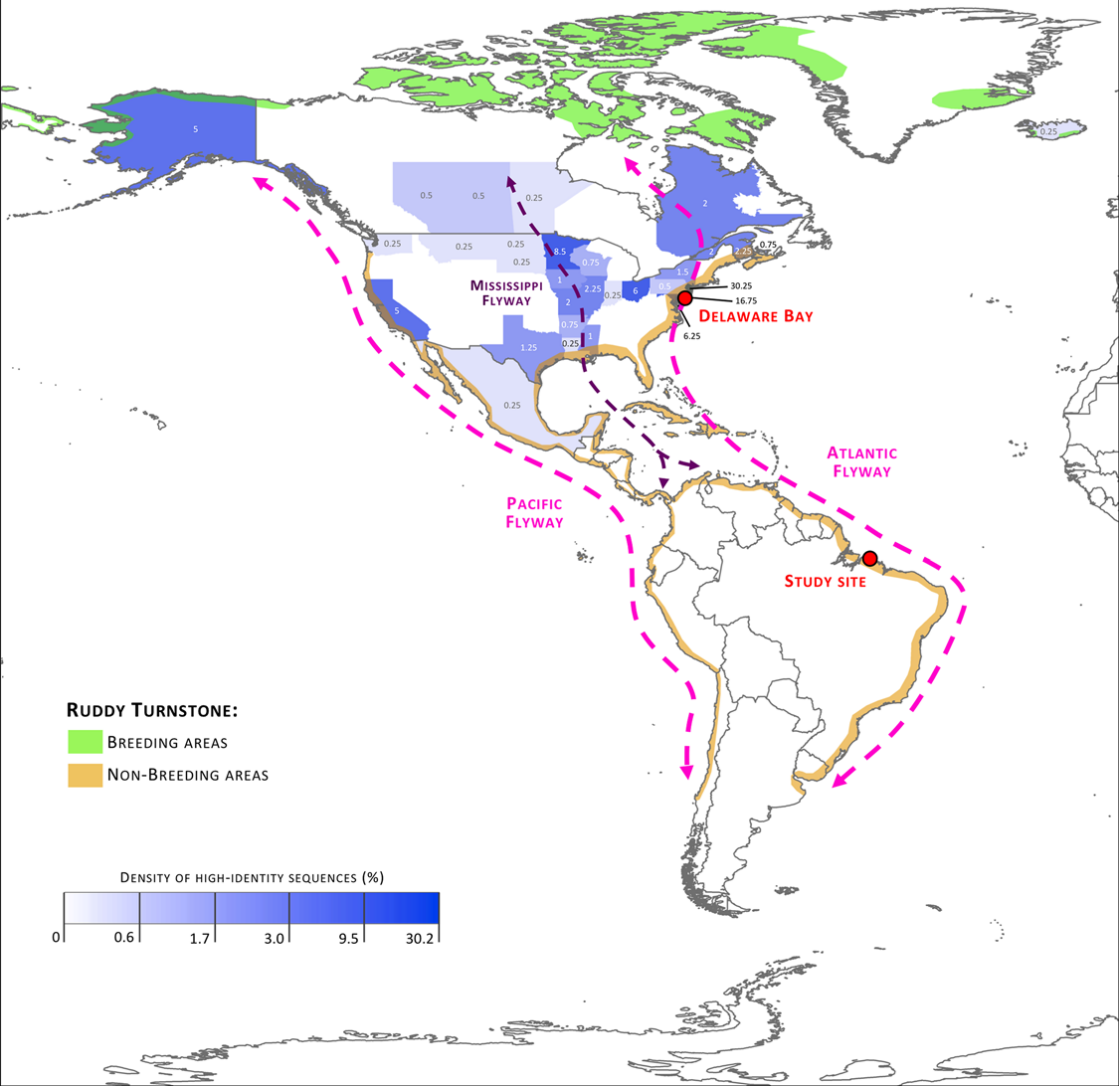
Distribution of high-identity sequences in relation to the study site and the natural distribution of ruddy turnstones and major flyways of migratory aquatic birds. High-identity density areas are represented in shades of blue, with more intense tones representing states or provinces where a higher proportion of high-identity sequences were identified (values represent the percentage of the 400 high-identity sequences identified).

Phylogenetic trees were obtained for each of the 8 segments of the H11N9 strains recovered from ruddy turnstones on the Brazilian Amazon coast (Figs 1 and 2). Detailed phylogenetic trees are provided as Supporting Information (S1–S8 Figs). Fig 3 represents the distribution of the high-identity sequences in relation to the natural distribution and migratory flyways of ruddy turnstones. Most high-identity sequences were from USA (93.25%) and Canada (6.25%), with only two sequences from other countries (Iceland and Mexico); more than half of these sequences were from AIV strains identified in the states near to the Delaware and Chesapeake Bays (New Jersey 30.25%, Delaware 16.75%, and Maryland 6.25%).

## Discussion

For all genes, the H11N9 strains analyzed in this study consistently clustered together within evolutionary branches of AIV that had been previously described from aquatic birds in North America. The only exceptions were the PA sequence of A/wild bird/Chile/1805/2008(H5N9) and the HA sequence of A/lesser black gull/Iceland/145/2010(H11N2), which both were closely related to the AIV strains in this study while also clustering within the broader North American lineage. In the case of the lesser black-backed gull (*Larus fuscus*), it is worth noting this species does not adhere to the general North American flyways since populations breeding in Iceland may winter along the eastern coast of the United States [39], which would explain why it was exposed to an AIV strain that bears resemblance to those of shorebirds in the Delaware Bay.

All genes of the H11N9 isolates from Canelas Island were closely related to AIV strains identified in aquatic birds in the Delaware Bay region both prior to and following 2008. In other words, this indicates that the virus was carried between North and South America with limited genetic re-assortment and mutation, a remarkable finding considering the more than 5000 km separating Canelas Island from Eastern USA. This is conceivable when the breadth of the migratory movements of some aquatic birds is considered, e.g. there have been documented cases of ruddy turnstones covering as much as 6200 km within 5 days [40].

Although there was an overall trend of sequence identity and phylogenetic clustering with AIV strains from shorebirds on the East coast of North America, a substantial genetic similarity was also found in relation to AIV strains from other aquatic birds in central USA and Canada, particularly Anseriformes (mallards, ducks and teals) along the Mississippi flyway. Ruddy turnstones are coastal birds and, as a result, are expected to utilize predominantly the coastal routes (Atlantic and Pacific flyways) with limited use of inland routes (Mississippi flyway) [37, 41]. Our findings therefore suggest that although ruddy turnstones on the Brazilian Amazon coast might not directly use the Mississippi flyway as a primary migratory route when traveling through North America, they are still exposed to AIV genes from other birds in that region, presumably through viral exchange in stop-over and wintering grounds shared by shorebirds and Anseriformes along the southern coast of North America.

The fact that the AIV strains from Canelas Island did not cluster with sequences of AIV isolates from South or Central America is not unexpected when considering the lack of publicly-available AIV sequences for those continents. Even though at the time the phylogenetic analyses in this study were conducted there were between 11.500 and 14.500 complete sequences of AIV for each segment in the IDB website, only between 12 and 23 were from South America (approximately 0.1% of the public database). The only Brazilian AIV for which complete gene sequences are available is A/semi palmated sandpiper/Brazil/43/1990(H2N1) [42]; this isolate had been obtained from a sample collected at Coroa do Avião, Pernambuco state [S. Krauss, unpubl. data], obtained 20 years earlier and approximately 1500 km from Canelas Island. The other nearest AIV sequences available for comparison are those obtained by Rimondi and colleagues [29] from Titicaca Lake, Bolivia, and by Karlsson and colleagues [28] from Villavicencio, Colombia, and both are approx. 3000 km from Canelas Island. The small number of sequences available for comparison in the region probably does not reflect a lower prevalence of AIV in birds in Brazil or South America, but instead results from a reduced sampling effort in the region [25]. Had other studies been conducted on the Brazilian Amazon coast, one could reasonably expect to find numerous other AIV strains showing genetic similarities to the H11N9 isolates analyzed in this study.

Ruddy turnstones are well known to have consistently higher prevalence of AIV than other shorebirds [13, 43, 44], and this species seems to play a key role in the persistence and spread of the virus at Delaware Bay [45]. There is speculation this could be related to the species’ habit of resting in wetlands and exposed mudflats or to the fact that, unlike most other shorebirds, ruddy turnstones will often feed on carrion and food waste [13]. Regardless of the mechanisms leading to the unique role of ruddy turnstones in the epidemiology of the virus, it is clearly a strategic target for AIV surveillance programs. For that purpose, three major wintering areas of ruddy turnstones [37] can be proposed as target areas for AIV surveillance in South America: (1) Northern Brazilian coast (Reentrâncias Maranhenses/Paraenses), (2) Suriname coast, and (3) Southwestern Peruvian coast (Paracas).

In conclusion, our findings corroborate the hypothesis that migratory flyways of aquatic birds play an important role in determining the genetic structure of AIV in the Western hemisphere, with a strong epidemiological connectivity among North and South Americas. Understanding this is relevant to anticipate the spread of AIV strains with potential impacts to human health, poultry industry or avian conservation in the region, and emphasizes the urgent need for additional studies on the occurrence and epidemiology of influenza viruses in South America.

## Supporting Information

S1 Fig Detailed phylogenetic tree for the PB2 gene. (PDF)

S2 Fig Detailed phylogenetic tree for the PB1 gene. (PDF)

S3 Fig Detailed phylogenetic tree for the PA gene. (PDF)

S4 Fig Detailed phylogenetic tree for the M gene. (PDF)

S5 Fig Detailed phylogenetic tree for the NP gene. (PDF)

S6 Fig Detailed phylogenetic tree for the NS gene. (PDF)

S7 Fig Detailed phylogenetic tree for the HA gene (subtype H11). (PDF)

S8 Fig Detailed phylogenetic tree for the NA gene (subtype N9). (PDF)
