# Supplementary figures and images for: Molecular Characterization of Subtype H11N9 Avian Influenza Virus Isolated from Shorebirds in Brazil
Source: PLoS One. 2015 Dec 21;10(12):e0145627. doi: 10.1371/journal.pone.0145627 (PMC4687026; doi:10.1371/journal.pone.0145627)

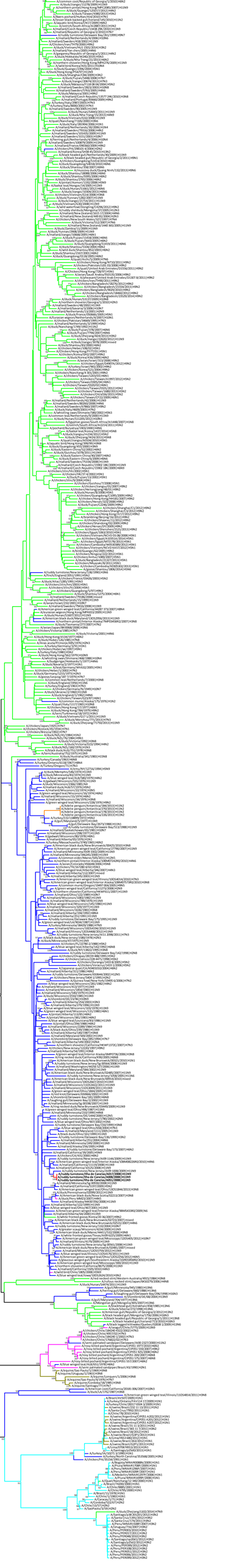

Supplement: S5 Fig — (PDF) [file pone.0145627.s005.pdf]

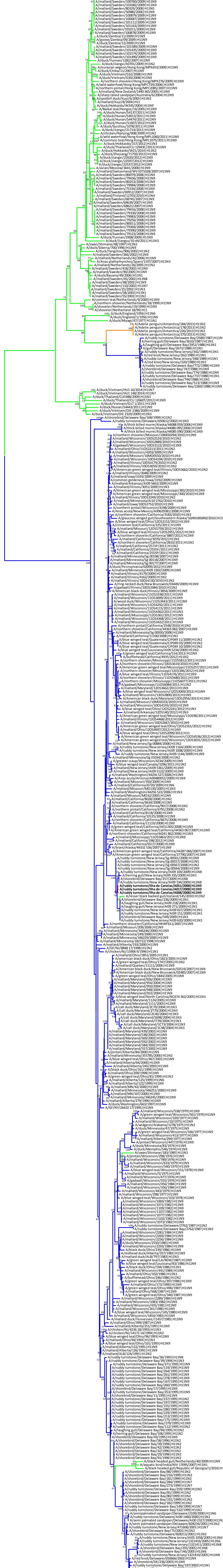

Supplement: S7 Fig — (PDF) [file pone.0145627.s007.pdf]
