# Supplementary material for: Molecular Characterization of Subtype H11N9 Avian Influenza Virus Isolated from Shorebirds in Brazil
Source: PLoS One. 2015 Dec 21;10(12):e0145627. doi: 10.1371/journal.pone.0145627 (PMC4687026; doi:10.1371/journal.pone.0145627)
Supplement: S6 Fig — (PDF) [file pone.0145627.s006.pdf]

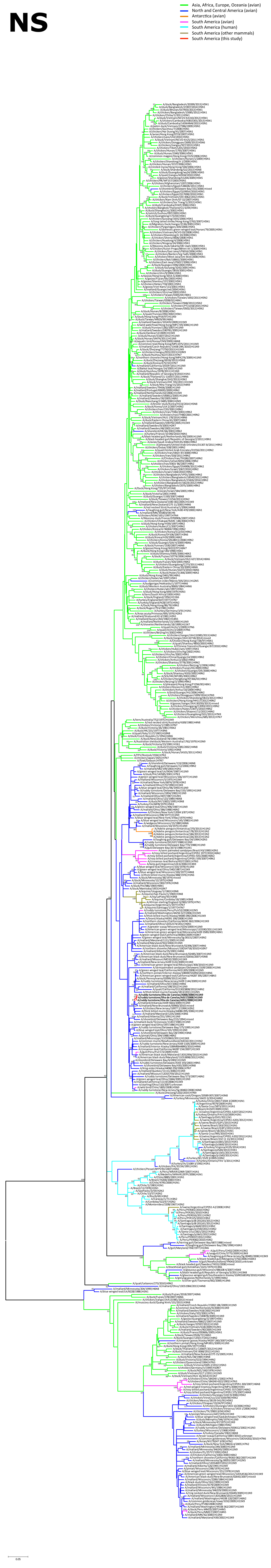
NS
Asia, Africa, Europe, Oceania (avian)
North and Central America (avian)
Antarctica (avian)
South America (avian)
South America (human)
South America (other mammals)
South America (this study)
0.05
